# An autosome-wide search using longitudinal data for loci linked to type 2 diabetes progression

**DOI:** 10.1186/1471-2156-4-S1-S8

**Published:** 2003-12-31

**Authors:** Gyungah Jun, Yeunjoo Song, Catherine M Stein, Sudha K Iyengar

**Affiliations:** 1 Department of Epidemiology and Biostatistics, Case Western Reserve University, 2500 MetroHealth Drive, Cleveland, Ohio, USA

**Keywords:** Type 2 diabetes, progression, longitudinal, genome-wide search, fasting glucose, model-free linkage analysis

## Abstract

A genome-wide screen was conducted for type 2 diabetes progression genes using measures of elevated fasting glucose levels as quantitative traits from the offspring enrolled in the Framingham Heart Study. We analyzed young (20–34 years) and old (≥ 35 years) subjects separately, using single-point and multipoint sibpair analysis, because of the possible differential impact of progression on the groups of interest. We observed significant linkage with change in fasting glucose levels on 1q25-32 (*p *= 5.21 × 10^-8^), 3p26.3-21.31 (*p *= 1 × 10^-11^), 8q23.1-24.13 (*p *= 2.94 × 10^-6^), 9p24.1-21.3 (*p *= 7 × 10^-7^), and 18p11.31-q22.1 (*p *< 10^-11^). The evidence for linkage on chromosomes 8 and 18 was consistent for the subset of study participants aged 43 through 55 years.

## Background

Type 2 diabetes mellitus is a common metabolic disorder affecting 4% of the world's adult population, and is defined by elevated blood glucose levels. The genetic basis of diabetes is more complex, involving both multiple interacting genes and environmental factors, which determine whether diabetes will develop and at what age [1,2]. The pathogenesis of type 2 diabetes requires defects in both β-cell function and insulin sensitivity. These abnormalities result in increased rates of glucose release as well as decreased glucose clearance from the circulation. Those who are at high risk of developing type 2 diabetes have diminished β-cell function at a time when many of them still have normal glucose tolerance [3,4]. Despite a genetic predisposition, diabetes may never manifest itself. Hyperglycemia, when it occurs, usually does so later in life (>50 years). Most linkage studies have examined fasting glucose levels as a quantitative trait or a dichotomized diabetes phenotype using cross-sectional data, and few longitudinal studies of diabetes progression exist.

We conducted a genome-wide scan of the rate of change in fasting glucose levels, as a surrogate for diabetes progression, using the data from offspring enrolled in the Framingham Heart Study to address two questions. First, we asked if the linkage signals identified by examining the average fasting glucose level during the 20-year interval or the rate of change in fasting glucose levels were the same. The hypothesis that drives this inquiry is that genes controlling average fasting glucose levels may be independent of those controlling the rate of change in fasting glucose. The second question is a corollary to the first, and relates to which cross-sectional measures should be used to derive the rate of change in fasting glucose. In this cohort, fasting glucose measurements were taken at five time points, 8.10, 4.32, 3.51, and 3.75 years apart, respectively. Thus, four rates (R1-R4) of change in fasting glucose can be calculated. If the rate and direction of change in fasting glucose is approximately equal in R1-R4, then use of a single measure or some function thereof will suffice. In contrast, if the rate and direction of change are sufficiently different, thereby influencing the sib-sib correlations for these measures, each rate, R1-R4, independently warrants closer examination.

We divided our participants into three groups, G1 (<20 years), G2 (20–34 years), and G3 (≥ 35 years) to contrast rates in groups of comparable age. The rationale behind this approach is that any locus that affects progression may act differently in different age groups, since age-at-onset is also one of the main features used to characterize type 1 diabetes, type 2 diabetes, and mature onset diabetes of the young. We anticipate that the regions we identified may harbor genes for progression to type 2 diabetes or to the prediabetic intermediate state.

## Methods

Subjects from the Framingham Heart Study (*N *= 1023 from the offspring cohort) provided in Problem 1 of the Genetic Analysis Workshop 13 were divided into three groups based on the age at baseline (<20 years (G1; *N *= 78), between 20–34 years (G2; *N *= 525), and ≥ 35 years (G3; *N *= 420)), after excluding individuals with incomplete data.

We initially conducted exploratory data analysis to determine the stage of diabetes in the subjects. We examined the number of individuals who had overt diabetes, defined as fasting glucose levels ≥ 126 mg/dl occurring at least twice in the ~20 year interval. Potential diabetes, defined as fasting glucose levels ≥ 126 mg/dl occurring at least once in the ~20 year interval, was also ascertained. To determine the age-specific stages of diabetes, we plotted changes in levels of mean fasting glucose stratified by age at baseline. We also examined the average fasting glucose levels at baseline and each following time point by subdividing the population into those with potential diabetes (≥ 26 mg/dl), those between 100–125 mg/dl, and normoglycemic individuals (<100 mg/dl). The rationale for this trifurcation was that those with clinical diabetes or impaired glucose tolerance might have undergone therapeutic interventions.

Fasting glucose levels were log-transformed prior to analysis to reduce skewness and to approximate normality. We used multiple linear regression models to examine the effects of sex, systolic blood pressure, and triglycerides, along with first order interactions of these effects, on fasting glucose levels at each time point. The progression rates were determined for each individual by calculating the difference in the residuals of fasting glucose levels between two observed time points. Four progression rates, estimated as differences in the standardized residuals divided by the age differences between time points 1 and 2 (R1), 2 and 3 (R2), 3 and 4 (R3), and 4 and 5 (R4), were estimated. Four newly derived phenotypes (R1-R4), and the mean of standardized residuals at five time points (20-year mean), were adjusted by a constant (× 100) and were analyzed as quantitative traits in all further analyses.

Sibling correlations for five derived phenotypes were estimated with the FCOR program in S.A.G.E. [5]. Sharing probabilities for identity by descent (IBD) were estimated at each marker and were interpolated at 2cM intervals using the GENIBD program [5]. We performed both single-point and multipoint linkage analyses on five quantitative traits (R1-R4, 20-year mean) in each stratum (G1-G3) using SIBPAL [5]. The weighted combination of squared trait difference and squared mean-corrected trait sum was used as the dependent variable in a Haseman-Elston regression model [6-8]. We selected the linkage evidence based on the genome-wide thresholds for mapping loci underlying complex traits [9]; the *P*-value for significant linkage is 2.2 × 10^-5 ^and the *P*-value for suggestive linkage is 7.4 × 10^-4^.

## Results

### Longitudinal data analysis

**Figure 1:**
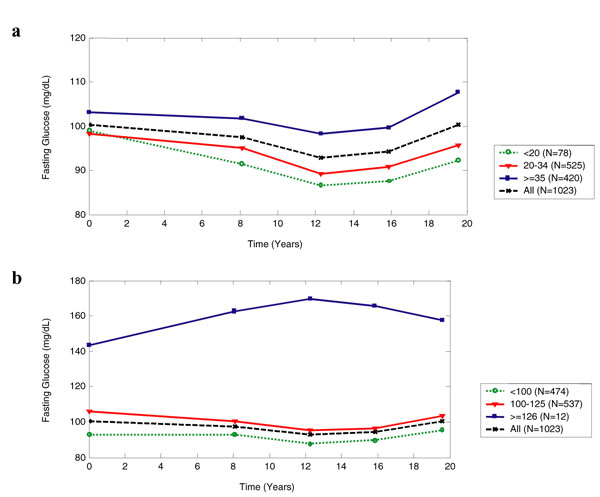
**Exploratory analysis of change in fasting glucose a. Change in levels of mean fasting glucose when stratified by age at baseline. **At baseline, the mean ages of grouped subjects were approximately 16 years for G1, 27 years for G2, and 43 years for G3, respectively. The mean of the fasting glucose levels over the five time points was 91.33 mg/dl in G1, 93.82 mg/dl in G2, and 102.06 mg/dl in G3, respectively. **b. Change in mean fasting glucose levels stratified by initial levels.**Fasting glucose levels were grouped at baseline as <100 mg/dl, 100–125 mg/dl, ≥ 126 mg/dl, and followed over all five time points.

Fasting glucose measurements for R1 were 8.10 years apart on average. Similarly, R2, R3, and R4 were based on measurements 4.32, 3.51, and 3.75 years apart, respectively. Average time between the first and fifth glucose measure was 19.68 years (Figure 1a). Unexpectedly, the average glucose levels declined with subjects' age from time point 1 to time point 3, and then increased again at the later ages. The trends were consistent in all age groups (Figure 1a).

We observed that a small subset of individuals (*N *= 12) with potentially overt diabetes remained diabetic at all time points (Figure 1b), although a small decline in mean fasting glucose was observed from time point 3 through 5. The fasting glucose levels in the <126 mg/dl groups, which constituted the majority of the sample, demonstrated a decrease in time points 2 and 3. We also estimated how many patients had type 2 diabetes. Within the G2 subgroup, only 8 subjects had overt diabetes, while another 18 had potential diabetes. Similarly, within the G3 subgroup, 56 and 32 individuals had overt and potential diabetes, respectively. The G1 subgroup had no subjects with overt diabetes or potential diabetes.

### Linkage analysis

There were 19, 259, and 293 informative sibpairs for G1, G2, and G3, respectively (Table 1). The mean size of sibships for G1, G2, and G3 is 1.28, 2.35, and 2.26, respectively (Table 1). The mean size of sibships between G2 and G3 was significantly different (*p *< 0.05); G2 had smaller sibships (Table 1). Due to the small sample size G1 was not included in further analysis (Table 1), nor were sibpairs whose members were split between two of these groups (e.g., G2 and G3). We observed that sex had a significant effect on fasting glucose levels and utilized sex-adjusted residuals for calculation of rates.

We examined correlations between siblings for mean fasting glucose and R1-R4 in G2 and G3 (Table 2). In general, we observed positive correlations for R3, R4, and 20-year mean glucose in G2, and for R1 and 20-year mean glucose in G3. Sibling correlations fluctuated from positive to negative to positive from R1-R4 in the G3 group. The correlations also fluctuated in G2, but not as significantly (Table 2).

**Figure 2:**
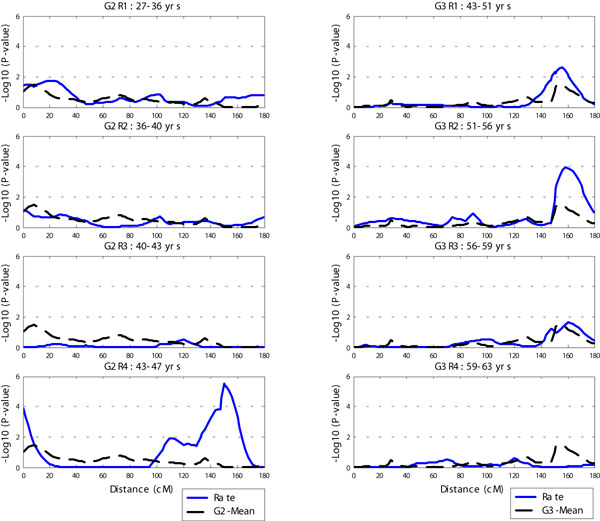
**Comparison of linkage results for young (G2) versus old (G3) subjects **Genetic distance (cM) is plotted for markers on chromosome 8 on the x-axis. R1-R4 in G2 was compared with R1-R4 in G3 (left and right; solid lines), and each contrasted with the mean glucose (dotted lines). The negative log_10 _(*P*-value) for linkage is plotted on the y-axis. *P*-values of 10^-2 ^and 10^-4 ^are shown as horizontal dotted lines perpendicular to the y-axis.

We focused on the linkage results from R4 in G2 subjects (age range: 43–47 years) who were approximately comparable in age to individuals in the R1 and R2 of G3 (age range: 43–56 years). Our analysis demonstrated evidence for linkage with R4 (G2), and R1 and R2 (G3), on chromosomes 8 and 18 (Table 3). We also observed significant evidence for linkage with R4 (G2) on chromosome 1, 3, and 9, and suggestive linkage on chromosomes 11 (Table 3). The linkage on chromosome 8 was consistent from ages 43 through 55 years (Figure 2). Suggestive linkage with 20-year mean glucose in G3 was obtained on chromosome 11 (Table 3).

## Discussion

Our analysis demonstrates that change in glucose levels over time is not linear in this cohort (Figures 1a and 1b). There are many explanations for the non-linear trend. Therefore, we focused on age-specific strata. We observed fluctuations in sibling correlations for G3, possibly because a greater number of subjects within this group are in the process of progressing to impaired glucose tolerance (IGT) or to type 2 diabetes (56 overt and 32 potential). However, only 12 of these remained diabetic across the entire 20-year period. The others cycled between normoglycemia, IGT, and diabetes. This oscillation, coupled with the overall decrease in the mean fasting glucose, at time points 2–4 probably lead to the difference in the sib-sib correlations in R1-R4 for G3. Similar factors may have affected the G2 sib-sib correlations, but to a lesser degree, because fewer diabetics were present in this group.

Diurnal variations and fluctuations in fasting glucose levels are part of the natural history of the disease [10,11]. To identify genes that influence progression, we need to capture the maximal sib-sib correlation associated with conversion of sibs from normoglycemia to IGT or from normoglycemia/IGT to diabetes. When analyzing a quantitative trait any increase from one time point to the next may be informative. Siblings appearing discordant for progression are some composite of those truly discordant, and those occasionally discordant due to fluctuations in glycemic status. These discrepancies will influence R1-R4 as well as the overall rate of change. Thus, estimation of overall rates of progression as the explanatory variable will only be useful if averaging the data captures the maximal sib-sib correlation described above.

In examining the sib-sib correlations, our conclusion is that the R3-R4 (G2 and G3) measurements may be our best approximations for the longitudinal measures. We recognize that the current data set is limited in this regard, and does not fully represent type 2 diabetes progression. To improve estimates of the rate of change in fasting glucose levels, each measurement must be adjusted for covariates, such as medication use or diet modification, which were unavailable to us.

If the effect of a progression locus is manifested at specific ages, then evidence for such loci may be obtained at specific age epochs with R4 in G2 being equivalent to R1-R2 in G3. We observed evidence for linkage at 18p11 in R4 for G2 and R1 for G3, and 18q21 in R4 for G2, as well as R2 for G3 (Table 3). Previous evidence for linkage on 18p11 has been reported in conjunction with obesity for type 2 diabetes [12]. The linkage on chromosome 18q is novel. Similarly, we observed evidence for linkage at 8q23-24 in R4 for G2 and in R1-R2 for G3 (Figure 2). Interestingly, the 8q23 region was reported as a locus for type 1 diabetes in a genetically isolated population from the Netherlands [13]. Two additional regions on chromosome 3 (~65 cM) and chromosome 11 (84–91 cM), previously identified from the Finnish population [14,15], also showed evidence of linkage (Table 3).

In a previous analysis of the Framingham Heart Study offspring cohort using variance components, a region on chromosome 1q (218 cM from pter; multipoint LOD = 2.33) was linked with the current fasting glucose levels [16]. We also observed a linkage signal on 1q in R4 (G2). This period is closest to the current time point described in the Framingham Heart Study, since it was calculated using data from the time points 4 and 5. Since this locus was observed both in the original Framingham data and in our analysis, it may control both mean glucose levels and progression.

## Conclusions

Our study provides further insights into how quantitative factors, such as rates of changes in fasting glucose levels, may play a role in the progression to type 2 diabetes. The resulting evidence from our analysis, particularly the linkage on chromosome 8 to the progression rate, and on chromosome 11 to the 20-year mean glucose, confirmed this hypothesis, and presents a compelling reason for further examination of these regions.

While our results are interesting, we conclude that future studies of diabetes progression need to refine measures to give consistent results between time points and groups. The source of fluctuations in the sib-sib correlations in G2 and G3 needs to be identified. For example, fasting glucose levels could be adjusted for additional covariates, specifically interventional therapy, as well as other variables that may enable us to reduce the intra-individual variability and minimize the oscillatory effect.

## Figures and Tables

**Table 1:** Descriptive statistics for sibships in G2 and G3 groups

**Groups**	**Sibships**	**Mean Size (Min, Max)**	**Sibpairs**
G1	61	1.28 ± 0.52 (1, 3)	19
G2	146	2.35 ± 0.61 (2, 5)	259
G3	98	2.76 ± 1.07 (2, 7)	293

**Table 2:** Sib-sib correlations for R1-R4 and mean glucose levels for G2 and G3 subjects

	**Rate**				
		
**Group**	**R1**	**R2**	**R3**	**R4**	**Mean Glucose**
G2	0.097	0.041	0.160	0.123	0.121
G3	0.150	-0.058	0.044	0.012	0.268

**Table 3:** Comparison of linkage results for subjects in G2 (R4) versus in G3 (R1 and R2)

**Ch #**	**Phenotype Group Rate: Age**	**Location**	**Distance (cM)**	**Most Significant Marker**	**Most Significant *P*-value**
**1**	R4G2: 43–47	q25 ~ q32	176 ~ 218	GATA48B01	**^A^5.21 × 10^-8^**
	R2G3: 51–55	p31.1 ~ p22.1	114 ~ 137	ATA29D04	0.0074
	G3 – Mean	p31.1 ~ p22.1	114 ~ 140	ATA2E04	0.0074
**3**	R4G2: 43–47	p26.3 ~ p21.31	6 ~ 71	GATA164B08	**1 × 10^-11^**
	G3 – Mean	p14.1 ~ q12.3	91 ~ 119	GATA128C02	0.0049
**8**	R4G2: 43–47	q23.1 ~ q24.13	119 ~ 135	GATA7G07	**2.94 × 10^-6^**
	R1G3: 43–51	q24.13 ~ q24.22	135 ~ 148	GATA21C12	0.0025
	R2G3: 51–55	q24.13 ~ q24.22	135 ~ 148	GATA21C12	*^B^1.06 × 10^-4^*
**9**	R4G2: 43–47	p24.1 ~ p21.3	14 ~ 44	GATA27A11	**7.0 × 10^-7^**
**11**	R4G2: 43–47	p15.5 ~ p15.2	2 ~ 21	GATA23F06	9.9 × 10^-4^
		q22.3 ~ q24.3	101 ~ 131	GATA23E06	*3.14 × 10^-4^*
	G2 – Mean	q12.1 ~ q13.5	56 ~ 76	GATA90D07	0.0090
	G3 – Mean Glucose	p14.1 ~ q12.3	91 ~ 119	GATA30G01	*6.6 × 10^-4^*
**18**	R4G2: 43–47	p11.31 ~ q22.1	13 ~ 107	GATA26C03	**< 10^-11^**
	R1G3: 43–51	p11.31	3 ~ 7	321xc9	0.007
	R2G3: 51–55	q21.2 ~ q21.32	80 ~ 89	ATA7D07	0.0059

^A^Bold *p*-values indicate statistically significant evidence for linkage. ^B^Italicized *p*-values are suggestive evidence for linkage by conventional criteria [9]; marker range in the table corresponds to *p*-values < 0.01.
